# Gastric Emptying and Intragastric Behavior of Breast Milk and Infant Formula in Lactating Mothers

**DOI:** 10.1093/jn/nxab295

**Published:** 2021-09-29

**Authors:** Guido Camps, Elise J M van Eijnatten, Glenn A A van Lieshout, Tim T Lambers, Paul A M Smeets

**Affiliations:** Division of Human Nutrition and Health, Wageningen University and Research, Wageningen, The Netherlands; Division of Human Nutrition and Health, Wageningen University and Research, Wageningen, The Netherlands; FrieslandCampina, Amersfoort, The Netherlands; FrieslandCampina, Amersfoort, The Netherlands; Division of Human Nutrition and Health, Wageningen University and Research, Wageningen, The Netherlands; Image Sciences Institute, University Medical Center Utrecht, Utrecht University, Utrecht, The Netherlands

**Keywords:** stomach emptying, stomach behavior, MRI, human milk, infant formula

## Abstract

**Background:**

When sufficient breast milk is not available, infant formula is often used as an alternative. As for digestion, gastric behavior of infant formula and breast milk have not been studied in detail.

**Objective:**

This study aimed to compare gastric emptying and intragastric behavior between breast milk and infant formula in vivo using MRI.

**Methods:**

In this randomized crossover study, 16 lactating mothers (age: 31.7 ± 2.9 y; time since giving birth: 9.3 ± 2 mo), underwent gastric MRI scans before and after consumption of 200 mL of infant formula or their own breast milk. MRI scans were performed after an overnight fast (baseline) and every 10 min up until 60 min following ingestion. Primary outcomes were gastric emptying measures and the secondary outcome was gastric layer volume over time. Differences between infant formula and breast milk in total gastric volume and layering volume were tested using linear mixed models.

**Results:**

Gastric emptying half-time was 5.1 min faster for breast milk than for infant formula (95% CI: –19.0 to 29.2) (*n* = 14). Within a subgroup (*n* = 12) with similar initial gastric volume (<20 mL difference), gastric emptying half-time was 20 min faster for breast milk (95% CI: 1.23–43.1). Top layer volume (*n* = 16) was 6.4 mL greater for infant formula than for breast milk (95% CI: 1.9–10.8). This effect is driven by t = 10 and t = 20 min postingestion.

**Conclusions:**

When taking initial gastric volume into account, breast milk emptied faster than infant formula in women, which is in line with previous findings in infants. Infant formula showed a significantly larger top layer volume in the first 20 min after ingestion. MRI in adults may find application in studies assessing gastric behavior of infant formula.

## Introduction

The golden standard to feed infants is breastfeeding, since breast milk is uniquely suited for the infant and has important health benefits (1, 2). However, for various reasons, parents can use infant formula as an alternative, which may have health consequences for the infant. For instance, breastfed infants gain less weight than formula-fed infants during their first years of life, which is associated with a reduced obesity risk up to adulthood (3). Differences in protein intake, protein metabolism, and metabolic signaling have been discussed as the underlying mechanisms (4, 5). Another contributor could be the fact that formula-fed infants develop a different feeding pattern compared with breastfed infants during their first months of life. Formula-fed infants increase their volume intake per feeding as the number of daily feedings decreases with age, whereas breastfed infants do not adjust their number of daily feedings and increase their volume intake to a lower extent than formula-fed infants (6). This results in an increased daily milk intake for formula-fed compared with breastfed infants (7). A possible explanation for the development of different feeding patterns might be related to the more active role of infants in determining intake when breastfeeding. In the case of bottle feeding, the mother may encourage her child to finish the bottle (8). On the other hand, it may be explained by compositional differences between breast milk and infant formula, since differences in the pattern of milk intake between breast milk and formula have also been demonstrated in studies that applied (bottle-fed) expressed milk (9). These compositional differences could lead to a different gastric emptying rate between breast milk and formula feeding and subsequently a different volume intake.

Gastric emptying of breast milk has been found to be faster than that of infant formula in infants (10–12). This has been shown using different methodologies, including scintigraphy and more recently, less invasive measurements like tracer breath analyses and ultrasound. For example, Van den Driessche et al. used a ^13^C-octanoic acid breath test in infants to show that gastric emptying of expressed breast milk was faster than that of infant formula (11). In addition, Ewer et al. used an ultrasound technique in preterm infants where gastric emptying of breast milk was twice as fast as infant formula (10). A potential explanation for these observed differences in gastric emptying are the compositional changes over the course of breastfeeding. The milk fat content, and therefore the caloric density, has been shown to gradually increase from relatively low in foremilk, to relatively high in hindmilk, whereas the composition of infant formula is homogenous during the feed (13–18). As a result, the foremilk that is initially emptied from the stomach into the intestines would have a relatively low lipid concentration and therefore lower caloric density compared with infant formula. Moreover, the gradual increase in lipid concentration with breastfeeding may contribute to layer formation (i.e., bottom low-fat and top high-fat layer) in the gastric compartment. The formation of a lipid layer in the stomach is mainly determined by emulsion stability under gastric digestive conditions and the result of coalescence and creaming of the milk fat globules (19). Homogenization could alter gastric emptying dynamics mainly as a result of disruption and rearrangement of the fat globule interface (20). A study by De Oliveira et al. with breast milk in preterm infants showed that homogenization of pasteurized breast milk slows down gastric emptying (21). The disruption and rearrangement of the fat globule interface results in increased gastric lipolysis, which generates free fatty acids. When these free fatty acids are released into the duodenum, after ingestion of homogenized breast milk, increased intestinal signaling slows down gastric emptying (22). Indeed, it has been shown in adults using MRI that a homogenous stable 15% oil emulsion empties slower than a nonstable emulsion (23), which is likely due to a decreased fat content of the lower layer in the nonstable emulsion. Therefore, we hypothesized that breast milk acts like a nonstable emulsion in the stomach resulting in a faster gastric emptying than infant formula, which acts, at least during the initial phase of gastric digestion, like a homogenous emulsion.

Thus far, studies have used methods such as scintigraphy, ultrasound, and ^13^C tracers to investigate gastric emptying of breast milk and infant formula. These methods can track gastric emptying but are less suitable for investigating intragastric processes like layer formation (24–26). Such processes can be visualized very well with MRI (27, 28). To the best of our knowledge, there are no in vivo studies on gastric emptying and gastric behavior of breast milk and infant formula using MRI. Due to ethical constraints, MRI cannot be used to assess gastric processes in healthy infants for research purposes. Therefore, this study aimed to compare gastric emptying and gastric behavior of breast milk and infant formula in lactating mothers to provide further insights into digestive differences between breast milk and infant formula.

## Methods

### Design

The study was a randomized crossover study with a balanced design in which lactating mothers underwent a total of 8 gastric MRI scans per session before and after consumption of 200 mL of their own breast milk or a stage 1 infant formula (intended for infants between 0 and 6 mo) (FrieslandCampina). Primary outcomes were gastric emptying half-time (GE t50) and gastric volume over time, the secondary outcome was gastric layer volume over time, and tertiary outcomes were subjective ratings (hunger, fullness, bloating, and nausea). The procedures followed were approved by the Medical Ethical Committee of Wageningen University in accordance with the Helsinki Declaration of 1975 as revised in 2013. This study was registered with the Dutch Trial Registry under numbers NTR7214 and NL7016. All participants signed informed consent.

### Participants

Lactating mothers were recruited in spring and summer of 2018 in the ZGV hospital in Ede and on the Wageningen university campus. Healthy, nonsmoking participants were included if they gave birth to a singleton ≥4 wk prior to participation. Participants were only included if they were lactating and had sufficient milk available for the experiment and feeding of their infant. Participants were excluded if they were lactating for >1 y, had bovine milk allergy or intolerance (self-reported), were lactose intolerant (self-reported), had gastric disorders or regular gastric complaints, made use of proton pump inhibitors or other gastric medication, had a contraindication to MRI scanning (including, but not limited to, pacemakers and defibrillators, intraorbital or intraocular metallic fragments, ferromagnetic implants, or were claustrophobic). The design and reporting of this study conforms to the Consolidated Standards of Reporting Trials (CONSORT) statement (29).

### Treatments

Treatments were 200 mL breast milk and 200 mL infant formula. A volume of 200 mL was chosen because of a limited availability of breast milk provided by mothers, given that their infants were still breastfed. The collection of breast milk was done over a maximum of 3 d before the day of consumption to ensure microbiological safety. Mothers were instructed to pool milk of different feedings and to collect the first 30 mL of expressed milk (foremilk), separately from the remaining milk (midstream and hindmilk) so that these could be consumed separately and in an approximate natural order as an infant would normally consume. They were asked to refrigerate the milk in a thermos bottle at 4°C until the day of the experiment. On the day that breast milk was consumed, participants were asked to bring 220 mL of their own breast milk stored in 2 cooled thermos bottles (60 mL foremilk and 160 mL remaining milk). From the 220 mL, 20 mL (10 mL of foremilk and 10 mL of remaining milk) was used for composition analysis, and 200 mL for consumption in the trial. Breast milk and infant formula were heated to 37°C using a water bath directly before consumption. The composition of infant formula and breast milk (foremilk, remaining fraction of the milk, and total) can be found in **Table 1**. Breast milk composition was determined using a commercially available human milk analyzer (MIRIS).

**TABLE 1:** Mean ± SD composition of infant formula and breast milk^1^

	Fat (g/100 mL)	Crude protein (g/100 mL)	Carbohydrate (g/100 mL)	Solids (g/100 mL)	Energy (kcal/100 mL)
Foremilk	2.9 ± 1.1	1 ± 0.25	8.0 ± 0.25	12.1 ± 1.0	62.7 ± 10.4
Remaining fraction of expressed milk^2^	3.9 ± 1.4	1 ± 0.1	7.9 ± 0.3	13 ± 1.2	72.3 ± 11.9
Total breast milk	3.7 ± 1.3	1 ± 0.1	7.9 ± 0.3	12.8 ± 1.2	69.9 ± 11.5
Infant formula	3.5	1.4	7.3	13	67

1 Values are mean ± SD, *n* = 13 (foremilk) or 16 (remaining fraction of expressed milk). Composition of total breast milk was calculated based on the volumes of foremilk (50 mL) and remaining fraction of expressed milk (150 mL). When no foremilk was available for analysis, the composition of the remaining fraction of expressed milk was used as total breast milk. One sample of infant formula was assayed once.

2 Remaining fraction of expressed milk is midstream and hindmilk.

### Study procedures

Gastric MRI scans were performed at baseline and at t = 3, 10, 20, 30, 40, 50, and 60 min after ingestion. Participants arrived after an overnight fast: eating was allowed until 22:00 on the day before the study and drinking water was allowed ≤1 h before the visit. Participants were scanned between 08:00 and 10:00 and were measured at the same time on both study days. After arrival, participants provided baseline appetite ratings and the baseline MRI scan was then performed. Subsequently, they consumed 200 mL breast milk or infant formula within 2 min. In the case of breast milk, the foremilk (50 mL) was drunk first after which the milk from the remaining expression (150 mL) was directly consumed. During the MRI session, participants verbally rated hunger, fullness, bloating, and nausea on a scale from 0 to 100 at each time point (30). There was ≥1 wk between the 2 visits.

### MRI

Participants were scanned in a supine position with the use of a 3-Tesla Siemens Verio MRI scanner (Siemens AG) using selected parameters: T_2_-weighted spin echo sequence (HASTE, 24 6-mm slices, 2.4 mm gap, 1.19 × 1.19 mm in-plane resolution, repetition time (TR): 850 ms, echo time (TE): 87 ms, flip angle: 112 degrees), with breath hold command on expiration to fixate the position of the diaphragm and the stomach. The duration of the scan was ∼18 s. A custom segmentation tool (made in MeVis lab, H. Kuijf, University Medical Centre Utrecht) was used to manually delineate total gastric content on every slice and to delineate the top layer. Volumes on each time point were calculated by multiplying the surface area of gastric content per slice with slice thickness, including gap distance, and summed over the total number of slices showing gastric content.

### Sample size

Based on pilot work using infant formula and our previous work (31–33), we estimated a within-patient SD in GE t50 of 15 mL, and a difference in means of 20 mL between the 2 interventions. Given an α of 0.025 and a power of 0.9, we calculated a minimal requirement of 14 participants. To accommodate for data loss due to (movement) artifacts, we included 16 participants to acquire ≥14 good-quality data sets.

The power calculation was done using software from: http://hedwig.mgh.harvard.edu/sample_size/js/js_crossover_quant.html.

### Statistical analysis

In order to estimate GE t50 an established linear-exponential model (34, 35) was used to fit a curve to the data of the gastric volume over time for the infant formula and breast milk sessions. This method works well for gastric content that increases due to gastric excretion in the early phase (lag phase) and afterwards empties almost linearly. Further analyses were performed in SPSS (version 22, IBM). GE t50 was compared between breast milk and infant formula with a paired t-test. GE t50 is a summary measure that is often used in other studies and therefore ensures the results can be compared with other studies. Differences between infant formula and breast milk in subjective ratings, layering volume, and total gastric volume were tested using linear mixed models with treatment, time, and treatment*time as fixed factors, participant as a random factor, and baseline values as covariates. Missing data was handled using a Maximum Likelihood estimation, Fisher's Least Significant Difference (LSD) posthoc tests were used to compare individual time points. In addition, Pearson correlation coefficients were calculated for gastric measures (GE t50, top layer volume at the first 30 min, and initial gastric content volume) and subjective ratings (hunger, bloating, fullness, and nausea). In addition, Pearson correlation coefficients were calculated for gastric measures and nutrient content (fat, crude protein, carbohydrate, total solids, energy, and true protein). For GE t50, we performed a posthoc analysis within a subgroup with similar initial gastric content. We observed that some participants had a relatively large difference in initial gastric volume between the 2 treatments. As a substantial amount of initial gastric juice will affect gastric pH after consumption and thereby affect digestion and gastric behavior, we also compared gastric emptying and behavior between the treatments within a subset of *n* = 12. As a cut-off point, we used 10% of the intervention load of 200 mL (20 mL), which led to the exclusion of 2 participants who had a relatively large difference of 40 and 42 mL. The significance level for all analyses was set at *P* = 0.05. Data are expressed as mean ± SD unless stated otherwise.

## Results

**FIGURE 1:**
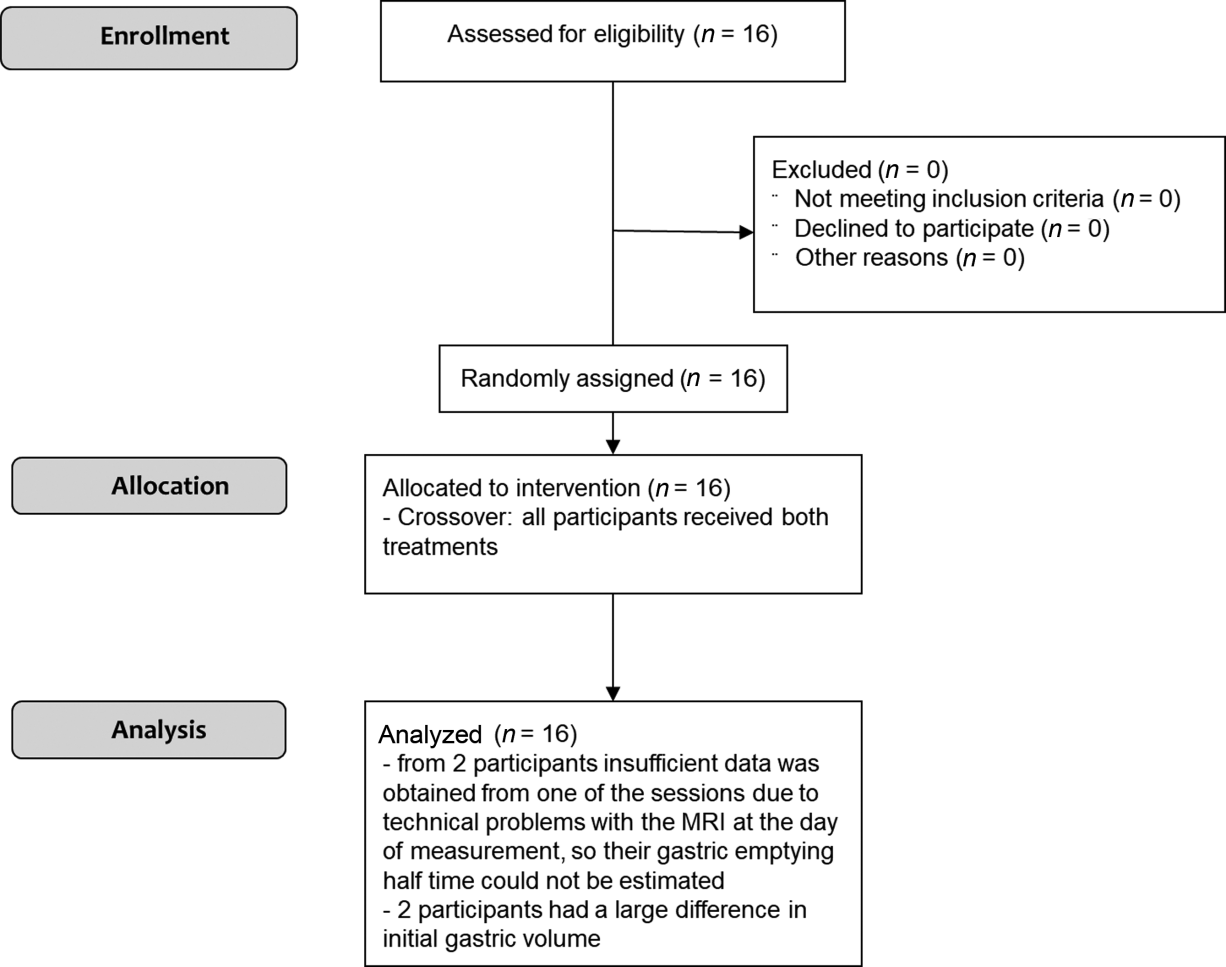
Study diagram.

Sixteen lactating mothers participated in the study (age: 31.7 ± 2.9 y, BMI: 22.6 ± 3.6 kg/m^2^, months since birth of the infant: 9.3 ± 2 mo). The macronutrient content of breast milk and infant formula in this study were similar, except for protein, which is higher in infant formula as expected (36). No data was lost due to (movement) artifacts, but insufficient data was obtained from 2 participants during 1 of the sessions due to technical problems with the MRI on the day of measurement. Data from all 16 participants were used for analysis and missing data was accounted for as described in the methods section, with the exception of GE t50, which could only be estimated for 14 participants. There were no relevant outliers seen in the data. The flow diagram of this study can be found in **Figure 1**.

**FIGURE 2:**
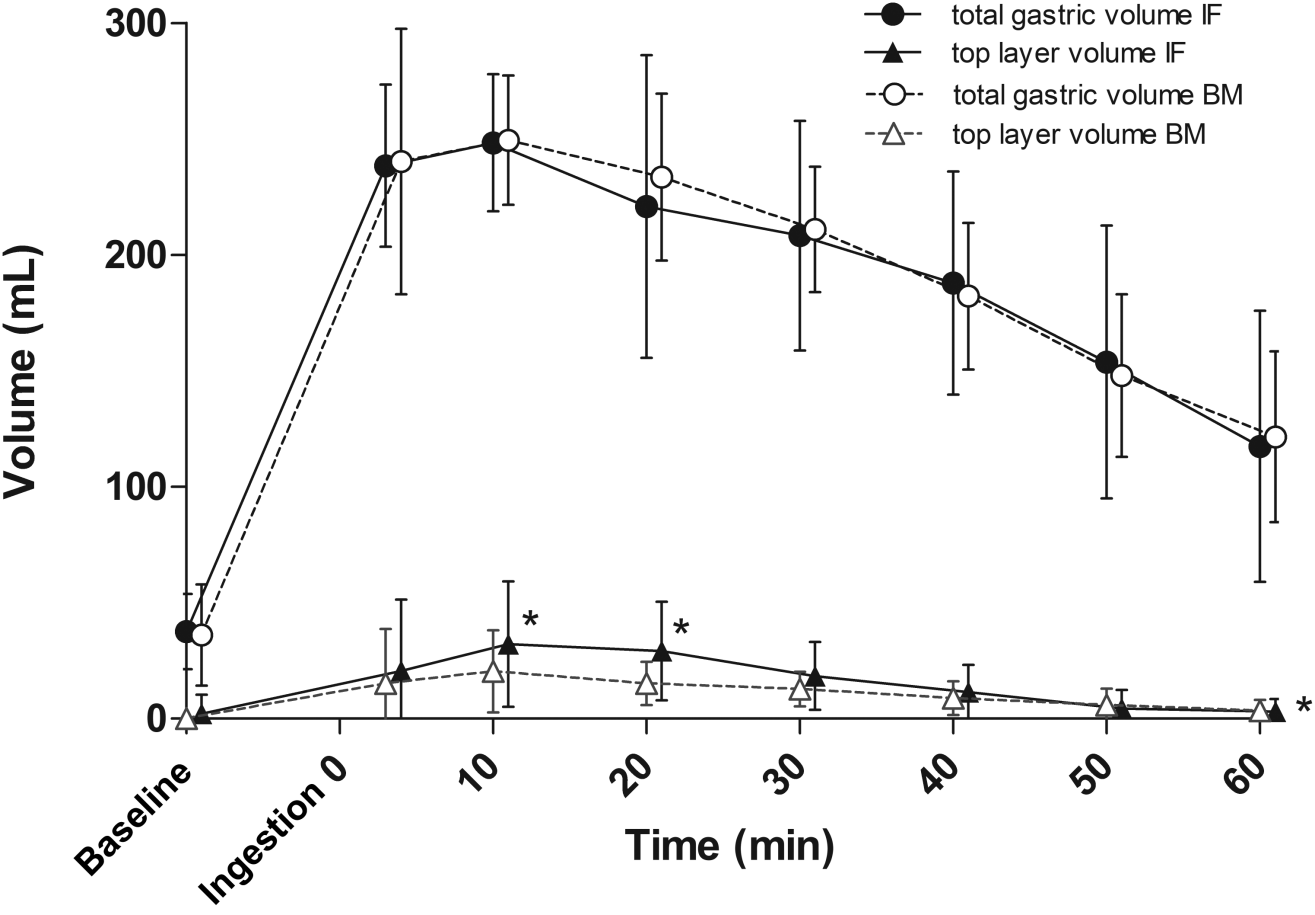
Gastric emptying for breast milk (BM) and infant formula (IF) displayed as gastric content and top layer over time in all participants. All values are mean ± SD (*n* = 16). *P <0.05, as analyzed with a mixed model analysis, showing a significant treatment effect for top layer volume and posthoc tests show that individual time points t = 10 and t = 20 min of top layer are larger for infant formula.

**FIGURE 3:**
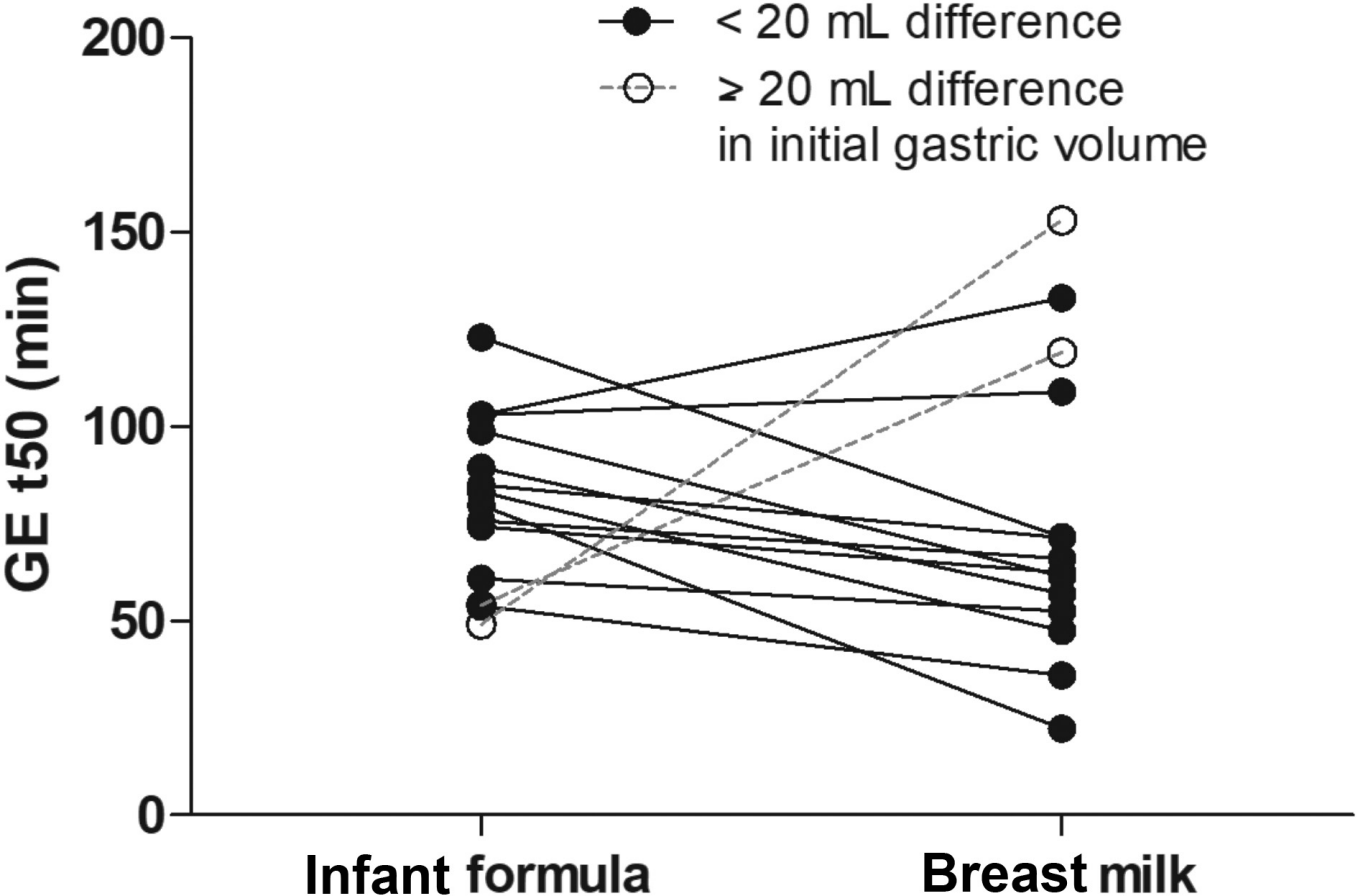
Gastric emptying half-times (GE t50) for infant formula and breast milk shown for all participants of which a curve could be fitted for both test sessions (*n* = 14). Participants with ≥20 mL difference in initial volume are shown as a dotted line. When excluding participants whose baseline gastric volume differed >20 mL between treatments, GE t50 was significantly lower for breast milk compared with infant formula.

GE t50 was 81.0 ± 21.6 min for infant formula and 75.9 ± 38.1 min for breast milk, treatment difference was 5.1 min (95% CI: –19.0 to 29.2). Gastric content volume over time did not significantly differ between treatments (**Figure 2**). The subset of participants (*n* = 12) with similar (<20 mL difference) initial gastric volumes shows a 20 min lower GE t50 for breast milk in comparison with infant formula (breast milk: 65.8 ± 29.9 min, infant formula: 85.8 ± 19.3 min, 95% CI: 1.23–43.1) (**Figure 3**).

However, the treatment effect of mean top layer volume over time was 6.4 mL greater for infant formula than for breast milk (breast milk: 10 ± 2.5 mL, infant formula: 17 ± 2.3 mL, 95% CI: 1.9–10.8). t-tests with LSD correction for multiple comparisons showed that this effect is driven by t = 10 and t = 20 min postingestion, since these timepoints differed significantly. An example of top layer visibility on an MRI scan is shown in **Supplemental Figure 1**.

To further explore the effect of gastric juice present at baseline, initial gastric volume was added as a covariate in the mixed model analyses of the complete dataset. As a result, top layer volume was no longer significantly different between breast milk and infant formula [breast milk: 15 ± 6.6 mL, infant formula: 20 ± 6.6 mL, treatment difference was 4.8 (95% CI: –14.9 to 24.4)].

### Subjective ratings

There were no significant differences in hunger, fullness, bloating, and nausea between the treatments. Bloating and nausea were largely absent. The graphs of the subjective ratings are shown in **Supplemental Figure 2**.

### Correlations

There was a significant positive correlation between bloating and top layer volume for breast milk (r = 0.59, *P* = 0.015). For infant formula, there was a significant positive correlation between top layer volume and bloating (r = 0.51, *P* = 0.044) and nausea (r = 0.66, *P* = 0.006). There were no significant correlations between fat, crude protein, carbohydrate, total solids, energy, true protein, and gastric measures (top layer volume, total content, and initial gastric volume).

## Discussion

This study compared gastric emptying and gastric behavior between breast milk and infant formula in lactating mothers. Gastric emptying and gastric layering of breast milk and infant formula were largely similar. However, when participants who had a large difference in initial gastric volumes (≥20 mL) between the treatments were excluded, breast milk emptied faster than infant formula. There was a significantly larger top layer volume for infant formula within the first 20 min after ingestion.

To the best of our knowledge this is the first study comparing gastric emptying and gastric behavior of breast milk and infant formula in adults using MRI. This method provides us with more insight but has 2 limitations, namely, using adults as participants for ethical reasons and the limited availability of breast milk to not hinder infant feeds. In contrast to previous studies involving infants, the combined dataset showed a similar gastric emptying for breast milk and infant formula. Studies using ^13^C octanoic acid breath testing, ultrasound, and scintigraphy have shown faster gastric emptying for breast milk than for infant formula (10–12, 26, 37). The different result for adults compared with infant studies can possibly be explained by differences in gastric physiology between adults and infants. The infant gastrointestinal system is still in development and therefore, gastric body size, gastric shape, and gastric secretions differ between adults and infants. In particular, the minimum gastric pH is higher in infants (3–5) than in adults (1–3.5) (38, 39), which influences activity of the digestive enzymes, gastric lipase and pepsin, and consequently the rate of digestion (40). Moreover, in our study, participants ingested 200 mL breast milk or infant formula, which is a relatively small amount for an adult stomach; this volume was chosen due to limited availability of breast milk. Consequently, by ingesting a relatively small volume, the initial gastric juice present at baseline likely had an impact on the pH of the ingested milk and thereby influenced gastric behavior. Indeed, we observed that the initial gastric volume affected the gastric emptying rate and gastric behavior of both breast milk and infant formula. When excluding participants whose baseline gastric volume differed >20 mL between treatments, GE t50 was significantly lower for breast milk compared with infant formula. This is in line with the discussed studies involving infants that showed a more rapid gastric emptying for breast milk than infant formula (10–12). We do not expect that water intake ≥1.5 h before the scan session will have an effect, since water usually takes <30 min to empty from the stomach (41).

Infant formula showed a significantly larger top layer than breast milk, despite the overall higher fat content and separate consumption of foremilk and the remaining fraction. The difference between the amount of fat in foremilk and the remaining expressed fraction was similar to that reported in another study (13). In the subset of 12 participants, no difference in top layer volume was shown between infant formula and breast milk. Although this observation is likely driven by the reduced sample size, this may suggest that initial gastric volume could be related to formation of the top layer, but no significant covariation was found. The rapid formation of a top layer in infant formula is in contrast to our hypothesis that infant formula would behave like a homogenous emulsion and therefore empty more slowly. However, it is still possible that the amount of lipids emptied in the early phase of gastric emptying may differ because of differences between the fat concentration of foremilk and infant formula. The larger formation of a top layer after ingestion of infant formula is most likely explained by differences in fat globule/emulsion stabilization between infant formula and breast milk. Breast milk naturally contains fat globules stabilized by a milk fat globule membrane (MFGM), which creates a stable oil-in-water emulsion. In infant formula, fat globules are predominantly stabilized by proteins due to homogenization and the resulting rearrangement of the fat globule interface due to surface active milk proteins, which results in a different gastric emulsion stability compared to MFGM-stabilized fat globules (42–44). In addition, protein denaturation in infant formula, as a result of the processing applied, can affect gastric emulsion stability as evident from in vitro studies (45). Indeed, both homogenization and heating of milk have been shown to influence phase separation and formation of a coagulum under in vitro gastric conditions, thereby affecting the gastric emptying of lipids (44). A possible explanation for the slower emptying of infant formula could be that heated, homogenized milk has been shown to form a softer coagulum that allows more of the coagulum to be emptied in the early phase of digestion. As a result, more nutrients were delivered to the intestine which could slow down gastric emptying. Overall, the observed difference in gastric layer formation between breast milk and formula is most likely explained by differences in fat globule stabilization.

We used MRI, which is a direct and more accurate method to assess gastric processes than indirect tracer-based methods (39). However, given the MRI measurements, this work could not be performed in infants because of ethical considerations, and adults were selected as a model. Our results suggest that gastric pH and the relatively large initial gastric volume, in comparison with the modest ingested volume of 200 mL, affected gastric emptying. To better mimic infant digestion in adults, a larger test volume is thus preferred and variations in baseline gastric volume should be taken into account when analyzing the data. In addition, MRI requires a supine position for scanning, which might slow gastric emptying (46). However, such effects might be minimal (47) and relative differences between treatments are expected to remain the same even if overall gastric emptying is slower. Moreover, infants will often digest their food in a supine position, thus from the perspective of infant nutrition and breastfeeding, the supine position was more natural in this study.

In conclusion, this study expands the existing knowledge about differences between breast milk and infant formula by providing novel insight into the gastric behavior and emptying of breast milk and infant formula using MRI. When differences in initial gastric volume between treatments were excluded, breast milk emptied faster than infant formula in line with previous observations in infants. Infant formula had a larger top layer within the first 20 minutes after ingestion, which is most likely explained by differences at the fat globule interface between breast milk and formula. Further elucidation of the processes underlying top layer differences between breast milk and infant formula and their implications for digestion and absorption is warranted.

## Supplementary Material

nxab295_Supplemental_Files

## Data Availability

Data will be available upon reasonable request to the authors.

## Notes

Supported by FrieslandCampina.

Author disclosures: GL and TL are employed at FrieslandCampin. All other authors report no conflicts of interest.

Supplemental Figures 1 and 2 are available from the “Supplementary data” link in the online posting of the article and from the same link in the online table of contents at https://academic.oup.com/jn/.

GC and EE contributed equally to this work.

Abbreviations used: GE t50, gastric emptying half-time, LSD, least significant difference; MFGM, milk fat globule membrane.
