# Supplementary material for: Gastric Emptying and Intragastric Behavior of Breast Milk and Infant Formula in Lactating Mothers
Source: J Nutr. 2021 Sep 29;151(12):3718–24. doi: 10.1093/jn/nxab295 (PMC8643590; doi:10.1093/jn/nxab295)
Supplement: nxab295_Supplemental_Files [file nxab295_supplemental_files.zip › Online Supplemental Material - figure legends 1.docx]

**Online Supplemental Material – Figure legends**

**Supplemental Figure 1.** Representative overview of all MRI slices showing gastric content from the fundus (top left) to the antrum (bottom right) at t = 20 min after ingestion of 200 mL breast milk (left) and infant formula (right). Top layer is marked in red, total content is marked in the combination of yellow and red.

**Supplemental Figure 2.** Graphs depicting mean ± SD for A hunger, B bloating, C fullness and D nausea over time after ingestion of  infant formula or breast milk (n=16)
