# Supplementary figures and images for: Gastric Emptying and Intragastric Behavior of Breast Milk and Infant Formula in Lactating Mothers
Source: J Nutr. 2021 Sep 29;151(12):3718–24. doi: 10.1093/jn/nxab295 (PMC8643590; doi:10.1093/jn/nxab295)

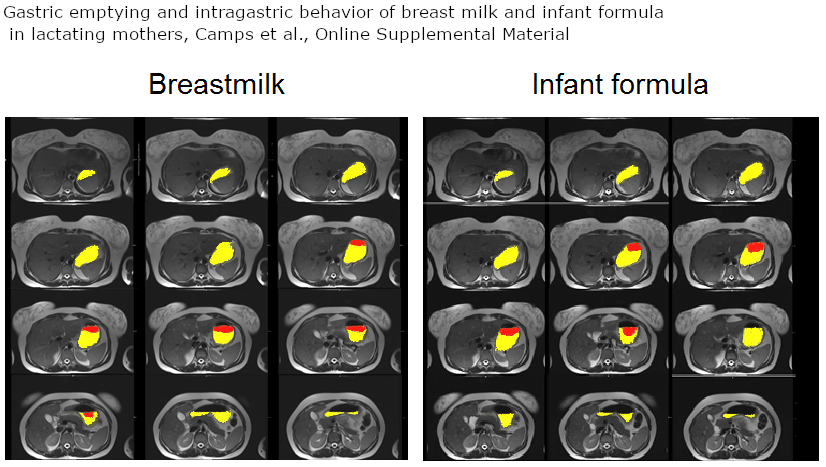

Supplement: nxab295_Supplemental_Files [file nxab295_supplemental_files.zip › Supplemental Figure 1 overview MRI slices.tiff]

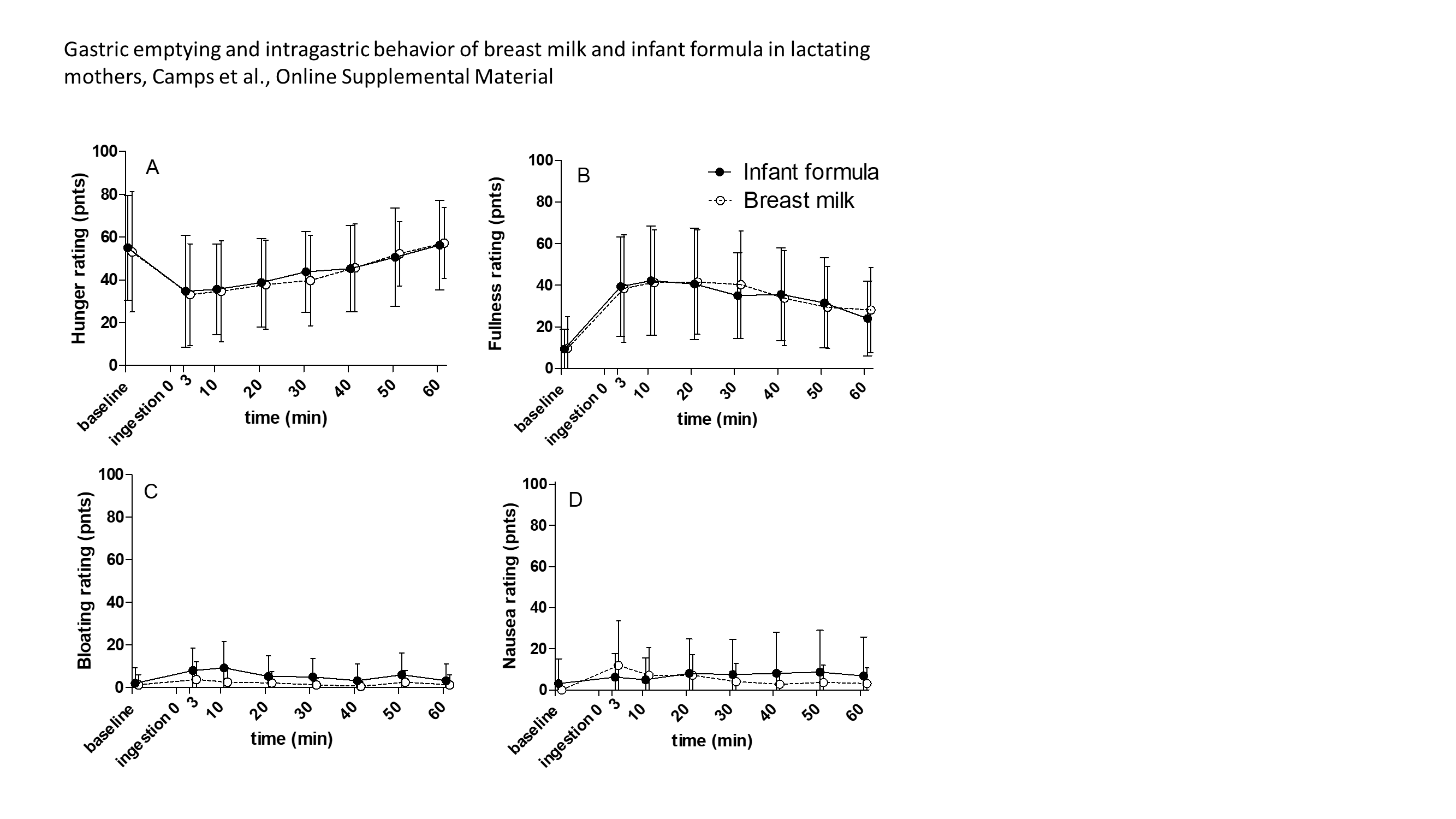

Supplement: nxab295_Supplemental_Files [file nxab295_supplemental_files.zip › Supplemental Figure 2 appetite ratings 1.tif]
